# From fossil-based to circular bioeconomy: a Swedish and Finnish pathway

**DOI:** 10.1007/s11356-025-36336-0

**Published:** 2025-04-04

**Authors:** Narasinha Shurpali, Yuan Li, Elina Tampio, Reijo Lappalainen, Ali Mohammadi, Maria Sandberg, Hem Raj Bhattarai, Ella Honkanen, Farinaz Ebrahimian, Ilmari Laaksonen, Lucia Blasco, Noora Jokinen, Venkatesh Govindrajan, Summaira Saghir, Vivek Narisetty, Karin Granström

**Affiliations:** 1 Grasslands and Sustainable Agriculture Group, Production Systems Unit, Natural Resources Institute Finland (Luke), Halolantie 31 A, 71750 Maaninka, Finland; 2 Present Address: Biorefineries and Bio-Based Fertilizers Group, Natural Resources Institute Finland (Luke), Latokartanonkaari 9, 00790 Helsinki, Finland; 3 Department of Technical Physics, University of Eastern Finland, Yliopistonranta 1 F, 70210 Kuopio, Finland; 4 Department of Engineering and Chemical Sciences, Karlstad University, 65188 Karlstad, Sweden; 5 Biorefineries and Bio-Based Fertilizers Group, Natural Resources Institute Finland (Luke), Tietotie 4, 31600 Jokioinen, Finland; 6 Moolec Science Limited, Innovation Centre, Gallows Hill, Warwick, CV34 6UW UK

**Keywords:** Forest residues, Biomass conversion, Biochar, Butanediol, Polymers, GHG emissions

## Abstract

The transition from a fossil-based economy to a circular bioeconomy is a critical challenge and opportunity in the face of global climate change. Sweden and Finland, with their abundant forest resources and strong commitment to sustainability, are well positioned to lead this transition. The WoodPro project exemplifies this effort by exploring innovative ways to valorize forest residues into high-value products such as 2,3-butanediol (2,3-BDO), biopolymers and hydrochar. This perspective outlines the project’s multidisciplinary approach, which integrates advanced bioprocessing technologies with dynamic system analysis to optimize the sustainability and economic feasibility of these biorefining pathways. We highlight the potential of these interconnected processes to reduce greenhouse gas emissions, close nutrient loops and stimulate rural development, while positioning the Nordic countries as global leaders in the circular bioeconomy. The insights gained from this project highlight the importance of holistic, systems-based approaches in achieving carbon neutrality and offer a model for similar transitions worldwide.

## Introduction

The circular bioeconomy offers a transformative alternative to the traditional fossil-based economy, which heavily relies on non-renewable resources and is a significant source of greenhouse gas (GHG) emissions (Kirchherr et al. 2023). While the transition to a circular bioeconomy presents considerable challenges, it also offers vast opportunities. Key challenges include the sustainable production of biomass, the development of cost-effective conversion technologies, the establishment of efficient supply chains and the promotion of a supportive regulatory framework (Tauseef Hassan et al. 2023). The opportunities are equally compelling: fostering innovation, generating employment, driving rural development and significantly reducing GHG emissions (Kirchherr et al. 2023; Tauseef Hassan et al. 2023).

Sustainable biomass production demands the adoption of environmentally friendly and socially responsible agricultural and forestry practices, taking into account biodiversity, soil quality, water usage and land use changes (Antar et al. 2021; Fahmy et al. 2020). Moreover, enhancing existing crops and developing new varieties can lead to more sustainable biomass yields (Antar et al. 2021; Fahmy et al. 2020). The optimization of conversion technologies, such as fermentation, enzymatic hydrolysis and thermochemical processes, is crucial for maximizing biomass utilization efficiency while minimizing its environmental impact (Bößner et al. 2021; Kataya et al. 2023). For instance, biomass rich in cellulose fibres, such as forest wastes and agricultural residues, can be converted to synthesize chemicals, biofuels and other bio-based products (Abolore et al. 2024).

Building efficient supply chains requires the integration of stakeholders across the production spectrum, from farmers to processors, distributors and consumers (Ansari and Kant 2017). Regional supply chains can stimulate local economies and reduce the environmental impact associated with long-distance transportation (Ansari and Kant 2017; Luthra et al. 2017). Crucially, this transition must be supported by a cohesive regulatory framework that promotes sustainable biomass production, technological advancement and market creation for bio-based products (Luthra et al. 2017). Policy coherence and active stakeholder participation are essential to successful transitioning to a circular bioeconomy (de Schutter et al. 2019; Luthra et al. 2017).

## Why is the shift to bioeconomy necessary?

The Nordic countries are already bearing the brunt of climate change, as evidenced by rising temperatures, melting glaciers and increased precipitation (Wirehn 2018). Since pre-industrial times, the average temperature in the Nordic region has increased by 1.5 °C, more than twice the global average (Rantanen et al. 2022). Projections indicate that temperatures could rise by an additional 1 to 4 °C by 2100 (Friedlingstein et al. 2010; Wirehn 2018). This warming trend, coupled with increased winter precipitation, has led to more frequent and severe floods, landslides, and increased erosion and sedimentation (Meier et al. 2022; Wirehn 2018). The anticipated melting of glaciers will further threaten freshwater availability, while shifts in ecosystems due to temperature changes will disrupt biodiversity, altering the distribution of flora and fauna (Meier et al. 2022; Wirehn 2018).

The impact of these climatic changes on soil health, plant life and human well-being is particularly concerning for the Nordic agricultural and forestry sectors (Wirehn 2018). Heatwaves, flooding and other extreme weather events are expected to exacerbate these challenges, necessitating urgent action. The European Union (EU) has set a target of achieving net-zero GHG emissions by 2050, with an interim goal of reducing emissions by 55% by 2030 compared to 1990 levels (European Commission 2023). The EU’s Adaptation Strategy outlines various measures to help member states cope with climate change, including enhancing infrastructure resilience, protecting biodiversity and reducing flood risks (European Commission, 2024). Additionally, the European Green Deal aims to transform the EU into a sustainable, climate-neutral economy by 2050 (European Commission, 2024).

In alignment with these EU initiatives, Finland has set an ambitious goal to achieve carbon neutrality by 2035, beginning with an 80% reduction in GHG emissions by 2030 relative to 2005 levels (Carbon Neutral Finland, 2021). Sweden, on the other hand, has targeted net-zero GHG emissions by 2045, with a goal to reduce emissions by at least 85% by 2045 compared to 1990 levels (Climate Policy Council, 2024). These targets emphasize the urgent need for a shift to a circular bioeconomy in the Nordic region.

## Availability of raw materials

The raw materials for bioeconomy are available in various forms such as wastes, residues and by-products. As per the EU Waste Framework Directive, wastes are materials with inelastic supply and no economic value. A waste is any substance or object which the holder discards or intends or is required to discard. Residues are secondary materials with inelastic supply and little economic value. Residues include agricultural, aquaculture, fisheries and forestry residues. They are directly derived from or generated by the mentioned sectors. In addition, processing residues are substances that are not the product that a production process directly seeks to produce. The production of the residue or substance is not the primary aim of the production process, and the process has not been deliberately modified to produce it. By-products are defined as a substance or object, resulting from a production process, the primary aim of which is not the production of that item. By-products can come from a wide range of business sectors and can have very different environmental impacts.

Forests cover 68.7% of Sweden’s land area and 74.2% of Finland’s, making these countries rich in renewable resources critical to the circular bioeconomy (Solheim et al. 2023). Both nations have a long-standing tradition in wood processing industries, including the production of paper, packaging and building materials. In 2020, Sweden produced 12 million tons of pulp, 9.3 million tons of paper and 18.5 million cubic metres of sawn timber, while Finland produced 7.7 million tons of pulp, 4.5 million tons of paper and 10.9 million cubic metres of sawn timber FAO (2020, 2020b; Nordic Forest Research, 2020). These figures underscore the significance of the forestry sector, which in 2021 contributed 0.6% to Sweden’s GDP and 1.7% to Finland’s GDP (Eurostat 2023).

Currently, forest-based residues such as bark, branches and sawdust are predominantly used for biofuel production, and pulp-and-paper-mill sludge is often incinerated (Lippke et al. 2011; Mohammadi et al. 2019a, 2019b; Raheem et al. 2018). However, these practices do not fully realize the potential of these materials. The European Green Deal, Circular Bioeconomy Package and the EU Taxonomy emphasize the importance of efficient recycling, utilization and valorization of biomass, including waste materials, to meet climate targets (European Commission, 2021). Sweden and Finland have set ambitious short-term targets towards carbon neutrality, which drive the innovation and development of greener forestry-based products to replace fossil-based alternatives and reduce GHG emissions.

Forest residues, including branches, tops, stumps, bark and sawdust left after logging or forest management operations, represent a vast and renewable resource for biorefineries within a circular bioeconomy (Mohammadi et al. 2019a; Ebrahimian and Ali Mohammadi 2023). In 2016, about 25% of Swedish energy was produced from a wide range of forestry feedstocks (Swedish Energy Agency, 2017), and solid wood fuels accounted for 15% of Finish energy consumption (Statistics Finland, 2017). In the USA, forest residues could contribute 368 million metric tons, or about 10% of the country’s energy demand (Tran et al., 2023). Beyond bioenergy, these residues can be converted into biochemicals, bioplastics and composite materials, offering significant values (Qian et al. 2024; Ebrahimian and Ali Mohammadi 2024).

However, the transition to valorizing forest residues is not without challenges. Sustainable forest management must balance the protection of biodiversity and ecosystem services with the economic and social well-being of local communities (Brás et al. 2024). Recent proposals by the EU Parliament, including the 2023 Renewable Energy Directive (REDIII), allow the use of wood residues for bioenergy while restricting the use of whole trees, emphasizing the need for efficient and sustainable supply chains to mitigate risks such as soil compaction, erosion and additional GHG emissions.

Paper mill waste, including sludge, bark, wood chips and wastepaper, presents another opportunity within the circular bioeconomy. The global production of paper mill waste is estimated at approximately 400 million tons annually (Bajpai 2015). Utilizing these wastes could potentially meet 5% of global transportation fuel needs and 10% of the global demand for chemicals (Gabrielli et al. 2023). Environmentally, converting paper mill sludge into biochar can reduce climate impacts and minimize soil toxicity associated with landfilling or low-energy incineration (Mohammadi et al. 2019a, 2019b). For instance, the residues of pulp and paper industry can serve as a substrate for enzyme production, particularly cellulase, xylanase and ligninase, due to their high lignocellulose content (Ghribi et al. 2016).

While these opportunities are promising, they come with challenges. The diverse composition of paper mill waste, including the presence of heavy metals and organic pollutants, can complicate its use in various bio-based applications (Mohammadi et al. 2019a, 2019b). Therefore, the development of efficient and sustainable extraction technologies is crucial for integrating these materials into the circular bioeconomy.

## Infrastructure needed for circular bioeconomy

### Research and development (R&D)

A robust R&D infrastructure is the backbone of the circular bioeconomy. Research institutes, laboratories and pilot plants play a critical role in developing new technologies, processes and products. Collaboration between these institutions and industry is essential to translate research findings into economically viable industrial solutions that can drive the circular bioeconomy forward.

### Biorefineries

Biorefineries have potential to play a pivotal role in the circular bioeconomy by transforming biomass into a variety of bioproducts, including fuels, plastics and chemicals. These facilities provide a platform for converting biological resources, including biowaste, into value-added products, thereby reducing GHG emissions and minimizing waste generation. However, the extent of circularity varies across different biorefineries, as some are still in the early stages of integrating new processing pathways and may operate alongside fossil-based production.

### Logistics and transportation

Efficient logistics and transportation infrastructure is vital for the movement of biological resources and products within the circular bioeconomy. This includes a network of roads, railways, ports, warehouses and distribution centres, all of which ensure that products are transported from producers to consumers efficiently and cost-effectively.

### Data management

The circular bioeconomy generates vast amounts of data that must be collected, stored and analysed to optimize resource use. Data infrastructure, including databases, data centres and cloud computing, is crucial for tracking the production and consumption of biological resources, analysing trends, and predicting future demand for bio-based products.

### Regulatory infrastructure

The circular bioeconomy is governed by a complex web of regulations and policies that oversee the production, use and trade of biological resources and products. Effective regulatory infrastructure, encompassing agencies and legal frameworks, is essential to ensure that the circular bioeconomy operates within ethical and legal boundaries. This infrastructure helps ensure sustainability and mitigate potential adverse environmental and social impacts.

## The WoodPro project: a holistic approach

**Fig. 1:**
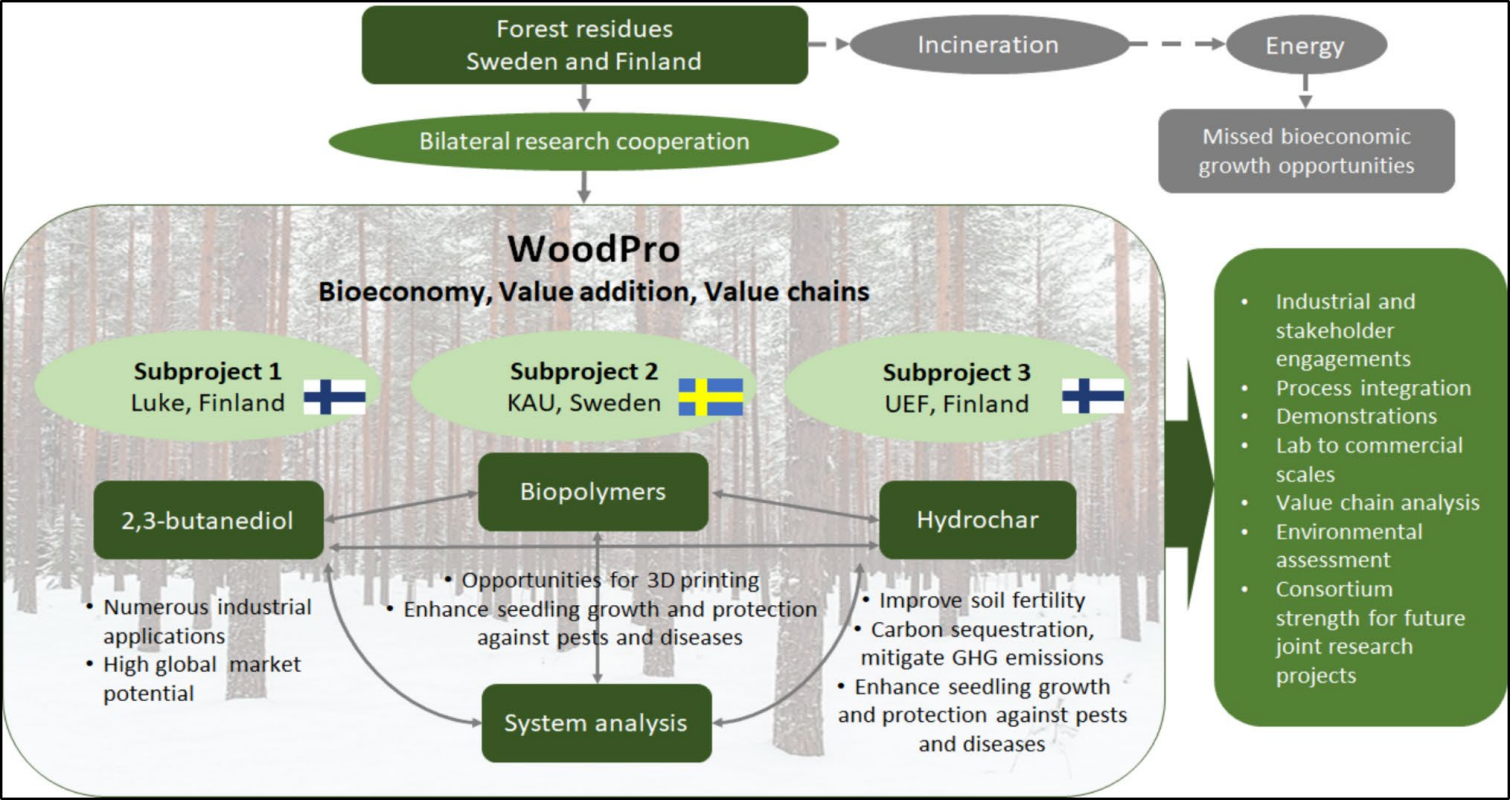
The WoodPro project scheme for the valorization of forest residues

Modern forestry increasingly embraces a high-value, low-waste model, and the WoodPro project exemplifies this approach through its novel, multidisciplinary and holistic process chain for adding value to forest residues. This project, a bilateral research collaboration between Sweden and Finland, is supported by funding from FORMAS in Sweden, the Academy of Finland, the Finnish Ministry of Agriculture and Forestry, and the Ahlström Foundation in Finland. The WoodPro project focuses on the integrated production of 2,3-butanediol (2,3-BDO), biopolymers and hydrochar, leveraging expertise from both countries (Fig. 1).

The methodologies and results from WoodPro are designed to expand the scope of technologies, products and end-uses over time, thereby supporting the transition to both a Green Economy and a Blue Economy, through applications in new sectors. In developing and assessing supply chains, multiple alternative and competing technologies may be available for each processing stage, leading to a variety of possible pathways. Selecting the best technology and finding the most optimal biorefining pathway (i.e. lowest environmental profile and highest profit) is a challenge. To address this complexity, multicriteria analysis is needed, and a system engineering design known as superstructure alongside mathematical modelling techniques (Elyasi et al. 2021) will be used to solve the problem. A key outcome of this project is the development of a ranking system for residue management scenarios, evaluated from both economic and environmental perspectives. This will enable the adoption of the most sustainable management strategies for forest residues. Additionally, the project’s approach, treating forest residues as part of an industrial ecosystem and a network of interconnected value chains, is expected to advance the adoption of future technologies that align with circular bioeconomy principles.

## BDO production

Carbohydrates present in forest residues can be microbially fermented into various chemicals using specialized bacterial strains. One such product, 2,3-BDO, offers a sustainable alternative to its petroleum-based counterpart (Song et al. 2019). While lignocellulosic materials like straw and oil palm have already been explored as substrates for 2,3-BDO synthesis, the optimization of forest-based residues for this purpose remains a key focus of the WoodPro project. In this context, the project examines spruce (*Picea abies*) and birch (*Betula pendula*) as primary feedstocks.

Key research questions include the following: What yield and purification levels can be achieved from the fermentative conversion of these forest residues, considering 2,3-BDO’s status as a platform chemical with numerous industrial applications? What levels of purification can be achieved using modern scalable techniques for various applications? Furthermore, can the residues from butanediol production—such as the lignin fraction, ethanol and volatile fatty acids (VFAs)—be further processed into hydrochar, biochar, solvents or biopolymers?

## Polyhydroxyalkanoates (PHA) production

Lignocellulosic feedstocks can be transformed into VFAs through acetogenic fermentation, which can subsequently be converted into platform chemicals (Song et al. 2019; Sun et al. 2021). These VFAs can also be utilized by specialized bacteria to produce polyhydroxyalkanoates (PHA), which serve as precursors for biodegradable plastics. While a variety of organic wastes have been explored as substrates for PHA (Bengtsson et al. 2008; Ganesh Saratale et al. 2021), the use of forest-sector residues remains underexplored (Bengtsson et al. 2008).

In the WoodPro project, mixed microbial cultures sourced from a forest industry wastewater treatment plant are employed to polymerize VFAs. The resulting PHA polymer is then extracted from the biomass, and its thermoplastic properties are evaluated. The primary research questions include the following: What yield and quality can be expected when producing PHA from residual streams derived from pulp and paper mill wastewater, 2,3-BDO production, and hydrochar production? What mix of VFAs is produced when these residues are fermented? And, will the PHA quality be suitable for manufacturing products like TubeSprout, a protective tube for forest seedlings during their first two years of growth?

## Hydro- or biochars production

Hydrothermal liquefaction/hydrothermal carbonization (HTL/HTC) and slow pyrolysis are promising methods for converting difficult-to-degrade lignocellulosic materials into hydrochar, biochar, liquids and gases. Hydrochars have demonstrated benefits for plant growth, enhancing the soil’s water and nutrient retention capacity (Eskandari et al. 2019). Additionally, both hydrochar and biochar have shown potential for short-term carbon sequestration and GHG mitigation (Smith 2016). Liquids produced from pyrolysis and HTL/HTC, often considered waste, can be repurposed as substrates for acidogenic bacteria in the production of PHAs (Behera et al. 2022).

**Fig. 2:**
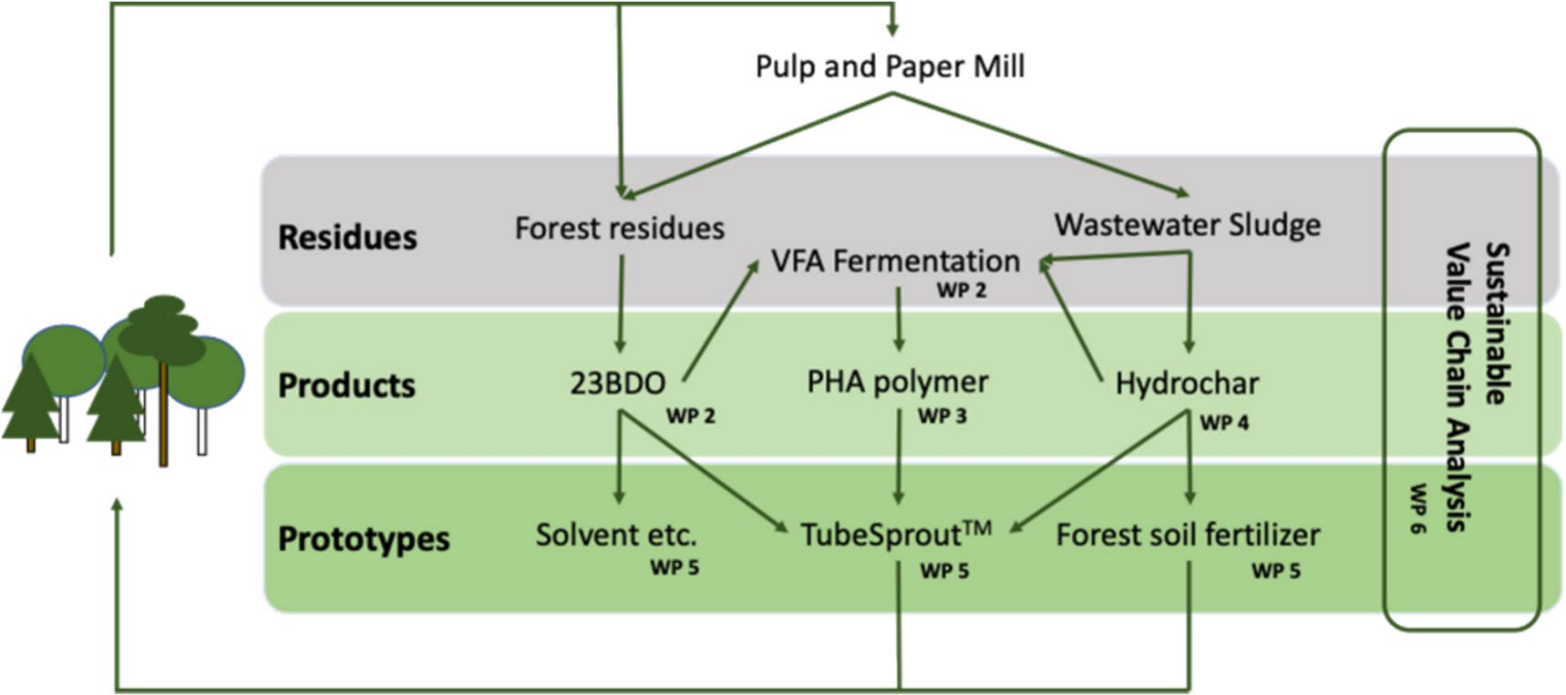
Work packages (WPs) in the WoodPro project and their interactions

Within the WoodPro project, birch bark residues are converted into hydrochar under conditions ranging from 200 to 400 °C and pressures between 5 and 25 MPa. Optimization involves the use of catalysts and solvents to improve yield and quality. Both hydrochar and liquid components are collected. Additionally, slow pyrolysis at a maximum temperature of 600 °C is used to produce biochar, suitable for applications such as barbeque fuel. Key research questions include the following: What yield and quality can be achieved in converting forest residues into carbon-sequestering hydrochar and/or biochar using HTC or slow pyrolysis? How effective are these hydrochars and biochars as soil amendments for tree seedlings, and how do they influence carbon flux in the soil? Are the purified liquid components of HTC and slow pyrolysis, such as VFAs and phenols, suitable for use as biopolymer ingredients (Fig. 2)?

## Sustainability of the value-added production

Multiple alternative technologies exist for each stage of the waste-to-value chain, presenting the challenge of selecting the most suitable one based on economic feasibility and environmental sustainability. This challenge can be addressed through multicriteria analysis (Rizwan et al. 2015). Additionally, system engineering design is recommended (Tan et al. 2014), specifically superstructure modelling, which employs mathematical techniques to assess potential pathways for producing 2,3-BDO, biopolymer and biochar from forest residues.

The WoodPro project seeks to answer key questions in this context: Are the production processes for 2,3-BDO, biopolymer and hydrochar sustainable strategies for managing forest residues from both environmental and economic perspectives? Among the technologies considered, which scenario represents the most sustainable biorefining pathway for valorizing forest residues into 2,3-BDO, biopolymer and hydrochar within a value chain framework (Fig. 2)?

## User cases

The central hypothesis of the WoodPro project is that forest-sector residues, typically viewed as low-value energy sources, can be effectively valorized into high-value products. This process aims to close nutrient loops and reduce GHG emissions. The medium-term objectives include stimulating the development of new value chains that can enhance job creation and support rural development, ultimately positioning Sweden and Finland as global leaders in the production and export of holistically sustainable bio-based products.

To evaluate the quality of PHA produced from forest residues, the project involves the creation of a TubeSprout™ prototype, which will be 3D printed or injection moulded. Its biodegradability will be tested in a microbiologically active environment. Additionally, TubeSprout™ will be fabricated using a hydrochar-biopolymer composite with the inclusion of 2,3-BDO.

The effectiveness of birch-derived hydrochar as a growth medium for tree seedlings will also be assessed. This hydrochar will be added in varying proportions (10–30%) to the standard peat growth medium used in plant nurseries. Previous experiments have demonstrated that incorporating 15–20% softwood-sourced hydrochar can reduce the need for synthetic fertilizers by half. Once the seedlings are ready for transplantation into the forest, hydrochar pellets can be applied to the soil to further enrich it.

Moreover, the suitability of birch-derived hydrochar as a soil amendment for agriculture will be tested on boreal legume grasslands, specifically Timothy and red clover, grown on mineral soil. Both soil physicochemical properties and GHG emissions will be measured to assess the overall impact.

## Sustainable value-chain analysis

An environmental life-cycle assessment (E-LCA) is employed to evaluate the environmental impacts of valorizing wood residues into bioproducts, compared to their conventional use as low-value energy resources. This assessment considers various factors such as climate change, human health and resource depletion. Central to the E-LCA is the development of a material flow analysis (MFA) model, which tracks the flow and stocks of materials within the proposed scenarios, connecting the sources, pathways and sinks of materials within the system.

In parallel, a techno-economic analysis (TEA) is conducted to estimate the net present value (NPV) of large-scale biorefinery projects. These analyses are integrated into a superstructure model, which is then used to identify the most sustainable valorization pathway for Nordic conditions, balancing environmental and economic considerations.

## A call to action

Achieving the vision of carbon neutrality demands a fundamental shift from fossil-based materials to biological alternatives. This transition requires an expansion of the feedstock base and the adoption of a biorefinery cascade-use approach. The WoodPro project exemplifies this strategy by using wood to initially produce 2,3-BDO, followed by the utilization of side-streams to produce biopolymers, biosolvents and hydrochar/biochar. Additionally, PHA is synthesized from residual streams generated during both 2,3-BDO and hydrochar production, while side-streams from hydrochar production are further utilized to create biopolymers.

A dynamic system analysis conducted within the project has revealed new insights into the potential of 2,3-BDO, biopolymer and hydrochar as an interconnected system, with quantifiable sustainability benefits. By developing superstructure models, WoodPro has identified and ranked the most sustainable pathways for valorizing forest residues into chemicals, biopolymers and hydrochar from a value chain perspective.

This holistic approach not only enhances the efficient use of forest resources but also significantly reduces waste and inefficiencies. It serves as a model for how the integration of various bioprocesses can support the transition to a sustainable circular bioeconomy. The successful implementation of such strategies will be crucial for realizing global carbon neutrality goals, and it is imperative that stakeholders across industries, governments and research institutions collaborate to scale these innovations and integrate them into broader economic frameworks.

## Data Availability

The current submission is of the type of Perspective/Opinion/Comments. It is not a research article. Hence, this submission does not warrant making data available.
